# First Report of the Stapled Mesh Stoma Reinforcement Technique in a Urologic Context

**DOI:** 10.1155/2014/294304

**Published:** 2014-10-22

**Authors:** Dwayne Tun Soong Chang, Isaac Andrew Thyer, John Oliver Larkin, Marina Helen Wallace, Dickon Hayne

**Affiliations:** ^1^Fremantle Hospital and Health Service, Alma Street, Fremantle, WA 6160, Australia; ^2^School of Surgery, The University of Western Australia, Crawley, WA 6009, Australia

## Abstract

Parastomal hernia is a common complication of ileal conduit formation. Mesh repair of parastomal hernia has lower rate of recurrence than nonmesh techniques but can be time-consuming to perform. The stapled mesh stoma reinforcement technique (SMART) is a novel method of rapidly constructing a reinforced stapled stoma. We report the first case utilising this technique in a urologic context. The procedure was performed on a middle-aged female with recurrent parastomal hernia of her ileal conduit. There were no perioperative complications. The resited stoma remained healthy and functioned normally. Longer term data is clearly desirable though this technique deserves consideration in the treatment of urologic parastomal hernias. This case demonstrates that SMART is an easy and convenient procedure for parastomal hernia repair.

## 1. Introduction

Parastomal hernia is a complication in up to 29% of patients after ileal conduit diversion [1]. Parastomal hernia recurrence rates after surgical repair such as nonmesh fascial suture repair, onlay mesh repair, sublay mesh repair, and underlay mesh repair were reported up to 57.6%, 14.8%, 7.9%, and 9.2%, respectively [2]. However, current mesh repair techniques for parastomal hernia can be time-consuming. The stapled mesh stoma reinforcement technique (SMART) is a novel and quick method of constructing a reinforced stapled stoma to reduce the risk of future parastomal hernia formation. We report the first case utilising this technique in a urologic context.

## 2. Materials and Methods

A 59-year-old female with an ileal conduit presented with fever, nausea, and bilateral flank and suprapubic pain over the past few days. This occurred on a background of an enlarging and increasingly symptomatic parastomal hernia at the ileal conduit. There was no passage of faeces or flatus for the past three days. She has a past history of recurrent parastomal hernia with previous open mesh stoma reinforcement repair 3 years before. On examination, there was generalized tenderness over her abdomen especially on both flanks and over the suprapubic region. A nonreducible and tender parastomal hernia was noted on the lateral side of the ileal conduit, with a positive cough impulse. Bowel sounds were normal. Her BMI was 34.8.

Abdominal X-ray revealed features suggestive of early or incomplete small bowel obstruction. A CT scan revealed bilateral hydronephrosis and hydroureter, enlargement of the parastomal hernia compared to previous scans, and dilation of the afferent limb of the ileal conduit in the intra-abdominal portion to the level of the parastomal hernia. A conduitogram confirmed ileal conduit obstruction at the level of the anterior abdominal wall. Her haemoglobin, white cell count, electrolyte levels, and renal function were all within normal limits. A diagnosis of an incarcerated parastomal hernia was made. Local repair of the parastomal hernia had been undertaken on a previous occasion; therefore we decided to perform a laparotomy to reduce the hernia and resite the conduit to the contralateral side.

**Figure 1:**
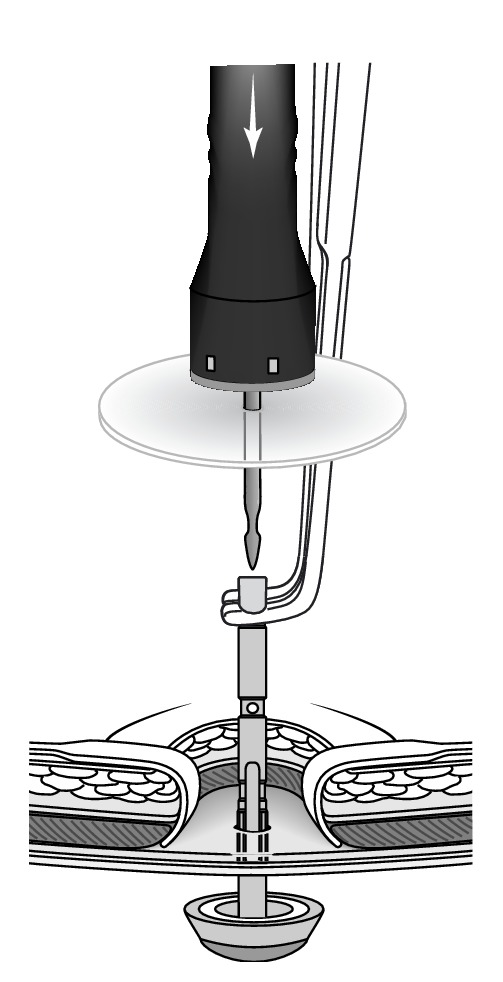
The circular stapler trocar was fitted with a mesh and engaged with the anvil. Reproduced with permission from Norman Williams © [3].

**Figure 2:**
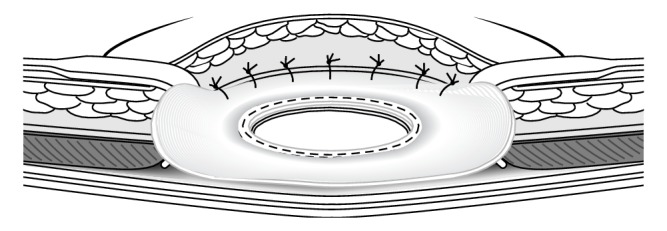
A mesh-reinforced stoma was constructed after the stapling device was fired and removed. The circumference of the mesh was sutured to the anterior rectus sheath. Reproduced with permission from Norman Williams © [3].

The ileal conduit was dissected free and mobilized, the parastomal hernia containing a loop of ileum was reduced, the hernial sac was mobilised, and the defect was repaired. A few centimetres of the distal end of the ileal conduit was resected back to healthy tissue. The loop of ileum within the hernia was well perfused and did not require resection. A new stoma aperture on the left side of the abdomen was fashioned; a cylinder of skin and fat was excised, and a cruciate incision was made on the anterior rectus sheath. A 25 mm CS Compact^TM^ EA circular stapler (Frankenman International Ltd., Hong Kong) was used to secure a ProLite Ultra^TM^ mesh (Atrium Medical Corporation, USA) to the staple line on the posterior rectus sheath. The anvil of the stapler was placed in the abdominal cavity and the shaft of the anvil grasped and delivered through the posterior rectus sheath. The stapler trocar was engaged with the anvil shaft and the stapler fired (Figure 1), leaving behind a reinforced stapled stoma consisting of the mesh, posterior rectus sheath, and peritoneum. The mesh circumference was secured to the anterior rectus sheath (Figure 2) and the ileal conduit passed through the reinforced stoma. The stoma was secured to the skin with undyed 3.0 Vicryl sutures. A standard stoma appliance was applied.

There were no perioperative complications and the resited stoma remained healthy and functioned normally. Renal function and electrolyte levels remained within normal limits. She resumed oral intake on Day 2 and was safe for discharge on Day 4. Follow up at 16 weeks showed a normal looking stoma with no evidence of recurrent herniation.

## 3. Discussion

The SMART procedure was first described by Williams et al. in 2011 [3]. We report the first utilisation of the SMART procedure in a urologic context. In our experience, the SMART procedure was highly convenient in constructing the stoma.

A formal cost-benefit analysis of implementation of SMART versus standard mesh repair was not performed; however the SMART procedure did leave a more neatly secured mesh which may translate into a potentially lower hernia recurrence rate. Formal data on this aspect is awaited from an ongoing randomised controlled trial (RCT) in the United Kingdom comparing SMART against standard stoma formation in general surgical patients. Results from this RCT, together with their cost-benefit analysis, will influence our decision to establish this as a standard technique at our institution.

The SMART procedure component of this operation took approximately a similar length of time as the traditional mesh reinforcement component for parastomal hernia repair. As our experience grows, particularly in thinner patients with fewer previous operations we expect this repair time to decrease significantly.

One issue is that mesh parastomal hernia repair of an ileal conduit exposed the mesh to risk of infection from leak of potentially infected urine. Fortunately, it is reassuring to note that the rate of mesh infection after mesh hernia repair in enterostomy cases is low, up to 2.7% in a systematic review, even with relatively greater bacterial load in the gut [4].

There are currently no published RCTs in the literature comparing SMART to other mesh techniques or to traditional methods of stoma formation. Clearly, more research with long term outcomes is required to clarify its potential advantages and disadvantages. Based on our limited experience, we believe that this technique deserves consideration in the treatment of ileal conduit parastomal hernias. Furthermore, there may be a role for performing the SMART technique electively during the formation of the initial ileal conduit to prevent parastomal hernia formation.

## 4. Conclusion

We have reported the first use of the SMART technique for stoma formation of an ileal conduit. Although SMART disposables are associated with extra cost, it is an easy procedure to perform and leaves a more neatly secured mesh that we believe is more likely to prevent recurrence of the parastomal hernia. More research is required to compare SMART to other parastomal hernia repair techniques especially in the field of urology.
